# Colectomy reconstruction for ulcerative colitis in Sweden and England: a multicenter prospective comparison between ileorectal anastomosis and ileal pouch-anal anastomosis after colectomy in patients with ulcerative colitis. (CRUISE-study)

**DOI:** 10.1186/s12893-023-01984-x

**Published:** 2023-04-21

**Authors:** Anton Risto, Caroline Nordenvall, Mohammed Deputy, Maria Hermanson, Ulrik Lindforss, Mattias Block, Omar Faiz, Pär Myrelid

**Affiliations:** 1 Division of Surgery, Department of Clinical and Experimental Medicine, Faculty of Health Sciences, University Hospital Linköping, 581 85 Linköping, Sweden; 2 Department of Surgery, County Council of Östergötland, Linköping, Sweden; 3 Department of Molecular Medicine and Surgery, Karolinska Institutet, Stockholm, Sweden; 4 Center for Digestive Disease, Division of Coloproctology, Karolinska University Hospital, Stockholm, Sweden; 5 St Mark’s Hospital and Academic Institute, Harrow, UK; 6 Department of Surgery and Cancer, Imperial College London, London, UK; 7 Department of Surgery, Institute of Clinical Sciences, Sahlgrenska Academy, University of Gothenburg, Gothenburg, Sweden; 8 Department of Surgery, Region Västra Götaland, Sahlgrenska University Hospital/Östra, Gothenburg, Sweden

**Keywords:** Ulcerative colitis, Reconstructive surgery, Ileal pouch anal anastomosis, Ileoanal anastomosis

## Abstract

**Background:**

There are no prospective trials comparing the two main reconstructive options after colectomy for Ulcerative colitis, ileal pouch anal anastomosis and ileorectal anastomosis. An attempt on a randomized controlled trial has been made but after receiving standardized information patients insisted on choosing operation themselves.

**Methods:**

Adult Ulcerative colitis patients subjected to colectomy eligible for both ileal pouch anastomosis and ileorectal anastomosis are asked to participate and after receiving standardized information the get to choose reconstructive method. Patients declining reconstruction or not considered eligible for both methods will be followed as controls. The CRUISE study is a prospective, non-randomized, multi-center, open-label, controlled trial on satisfaction, QoL, function, and complications between ileal pouch anal anastomosis and ileorectal anastomosis.

**Discussion:**

Reconstruction after colectomy is a morbidity-associated as well as a resource-intensive activity with the sole purpose of enhancing function, QoL and patient satisfaction. The aim of this study is to provide the best possible information on the risks and benefits of each reconstructive treatment.

**Trial registration:**

ClinicalTrials.gov Identifier: NCT05628701

## Introduction

Ulcerative Colitis (UC) is a chronic inflammatory bowel disease (IBD) restricted to the mucosa of the rectum and colon [1, 2]. The corner stone of UC treatment is pharmacological [3]. For about 10–15% of patients with UC medical treatment is not sufficient to induce/maintain remission or dysplasia/cancer occurs, and a colectomy will eventually be required [4, 5]. Although there are instances where a proctocolectomy with concomitant reconstruction is performed the recommended strategy for UC is usually a subtotal colectomy and reconstruction at a later stage [6, 7]. In a subtotal colectomy the distal colon is divided just above the promontory of the sacrum leaving a rectal remnant. The colon is removed and the terminal ileum is brought out through the abdominal wall as a stoma.

After subtotal colectomy there are four available options for patients. A reasonable option is to not proceed for further surgery and leave the rectum in place—the patient will live with a permanent ileostomy. Another option is the ileal pouch anal anastomosis (IPAA) where the rectum is removed and a pouch is created from the distal part of the ileum which is stapled or sutured to the anal canal or just above [8]. The IPAA is considered the gold standard reconstruction after subtotal colectomy [9]. A third option is the ileorectal anastomosis (IRA) where the rectum is left in place and the terminal ileum is stapled or handsewn to the top of the rectal remnant [10]. Then, for the rare instances that the patient is not suitable for either IPAA or IRA but still wants to avoid a stoma appliance, the Kock pouch is an option. The Kock pouch is a continent ileostomy that is actively emptied with a tube through the abdominal wall [11]. However, the Kock pouch is not commonly performed compared to the above options and will not be evaluated in this study. In a nationwide register study comparing Sweden and England only 46% and 33% of patients treated with colectomy received any reconstruction, respectively [12]. Whether the patients were offered reconstruction is unknown.

There are no randomized controlled trials (RCT) comparing IRA to IPAA. In a decision model using a Markov simulation in comparing IRA with IPAA in UC, the former was the preferred treatment option when quality-adjusted life-years were the outcome, while higher life-years was true for the latter [13]. An attempt of an RCT was conducted in Sweden led by Linköping starting late 2000 but after receiving adequate and standardized preoperative information the patients insisted on choosing surgical method, with a similar spread between the two options, and refused to be randomized. Therefore, the study was stopped. No protocol or results where, unfortunately, ever published from that RCT.

## Method/design

### Study objectives

To compare, in a prospective setting, patient satisfaction, QoL, function, and complications between IRA and IPAA and permanent stoma among patients with UC subjected to subtotal colectomy.

This study aims to answer what type of reconstruction, if any, UC-patients asks for following a colectomy, their satisfaction with the treatment, postoperative function and QoL. The results may have a large impact on future treatment recommendations.

### Study design

The CRUISE study is a prospective, non-randomized, non-blinded, multi-center, controlled trial on satisfaction, QoL, function, and complications between IRA and IPAA and permanent stoma among adult UC patients subjected to subtotal colectomy. All adult UC patients scheduled for a subtotal colectomy will be asked for informed consent. The patients will then be presented standardized written and video recorded information on the available reconstructive options. If the patient meets the inclusion criteria, their preferred choice of IRA or IPAA will assign them to one of the study arms. Patients that do not meet the inclusion criteria or fulfill any of the exclusion criteria (e.g. not suitable for an IRA) or refrain reconstruction will be asked to participate as controls.

### Endpoints

The primary endpoint is satisfaction with the choice of reconstructive method or permanent stoma. Secondary endpoints are QoL, sexual function, bowel function and complications.

### Study population

The study population consists of all adult UC patients subjected to subtotal colectomy and eligible for both IRA and IPAA presenting at any of the participating centers.

**Fig. 1:**
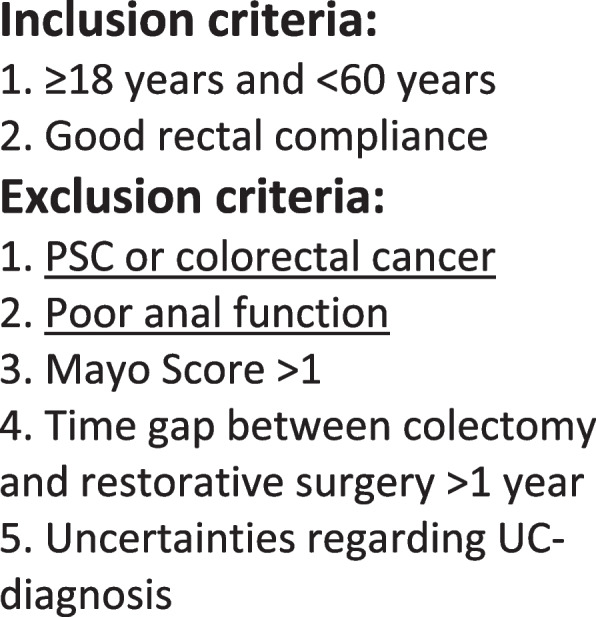
Inclusion and Exclusion criteria

Inclusion criteria are patients with UC aged between 18 and 60, scheduled for or have previously undergone subtotal colectomy and ileostomy. Patients should have sufficient rectal compliance and controllable inflammation in the rectal using topical 5-ASA only (Fig. 1).

Exclusion criteria are rectal inflammation of Mayo Score > 1 [14], poor sphincter function, perianal disease, uncertainty regarding UC diagnosis (IBD-U or possible Crohn’s disease), previous colorectal cancer or severe dysplasia (reported to the cancer registry), PSC diagnosis or > 2 year since subtotal colectomy (Fig. 1).

### Sample size

The only available study on patient satisfaction, our primary outcome, between IRA and IPAA reports 98% and 88% satisfaction for the methods respectively [15]. However, they only asked the IRA patients that still had their IRA in place after a mean of 11 years making it a little difficult to interpret. Another study compared QALYs between IRA and IPAA and reports a mean of 33.42 for IRA and a mean of 31.57 for IPAA [13]. With an estimated 3 SD (it was 2.8 for IRA and 4.5 for IPAA) and the significance level 0.05 and 80% power that difference would require 43 patients in each group to demonstrate a difference. Due to the mentioned shortcomings of the study comparing satisfaction we regard the latter study more relevant and decided to aim at a minimum of 50 patients in each group. Deliberately a little over the power estimation to avoid any effect of possible loss to follow-up.

### Participating centers

Patients will be enrolled from three tertiary referral centers in Sweden (Linköping University hospital, Linköping, Sweden; Karolinska University hospital, Stockholm, Sweden; Sahlgrenska (Östra) University hospital, Gothenburg, Sweden) and one tertiary referral center in the UK (St Mark’s Hospital and Academic Institute, Harrow, UK).

### Ethics

The study was approved by the regional ethics review board in Stockholm, Sweden (Dnr: 2017/124–31/2, 2018/2224–32) and The London Brent Research Ethics Committee (REC), UK (reference number: 18/LO/1190). The study is conducted in accordance with the Helsinki declaration and good clinical practice.

### Trial registration

The protocol is registered and published at ClinicalTrials.gov Identifier: NCT05628701.

### Study outline

#### Recruitment

**Fig. 2:**
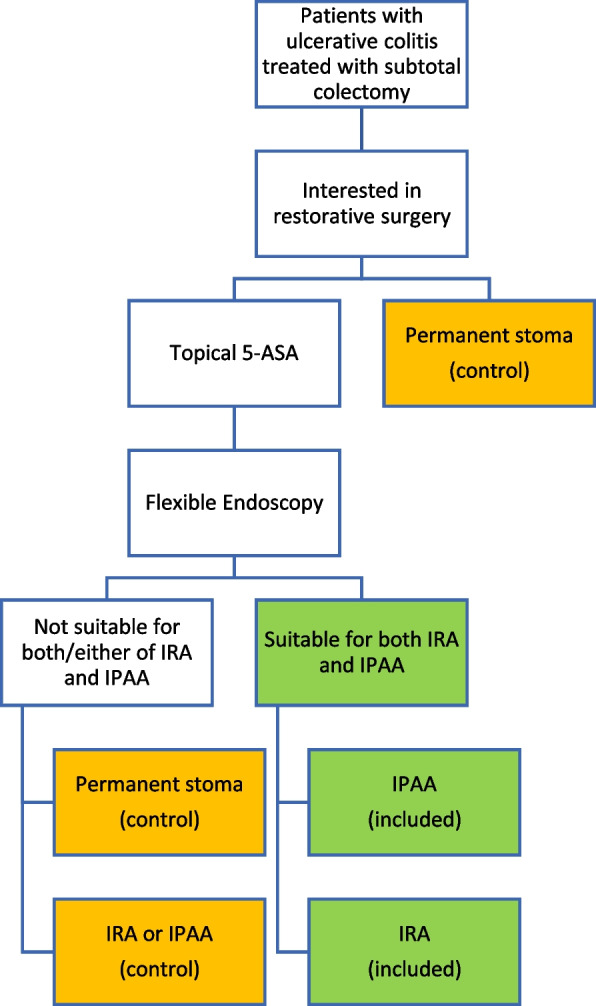
Flow chart describing the selection of patients eligible for the study (green). The other patients (yellow) will be asked to participate as controls

After colectomy, patients will receive standardized written as well as oral information from a consultant regarding the collection of QoL measurements, functional scores and the different treatment modalities before being asked for consent to participate in the study. The patient will be prescribed topical 5-ASA according to local principles, usually 500–1000 mg twice daily. 3–6 months after the colectomy patients will be subjected to rectal endoscopic examination to assess rectal inflammation and compliance and sphincter function. Inflammation is assessed by Mayo score while the assessment of rectal compliance and sphincter function is based on patient history and the subjective evaluation by the responsible surgeon. Based on these factors it is determined if the patient is eligible for IRA as well as IPAA. Patients that are deemed eligible for both reconstructions, and otherwise fulfill inclusion criteria, will be analyzed in the study arms and will henceforth be referred to as the study arms in contrast to the controls (Fig. 2).

#### IRA

The IRA can be performed both as an open or laparoscopic procedure, of which the latter is more common and preferred in modern practice. The ileostomy is closed, and the neoterminal ileum is in most cases anastomosed to the tip of the rectal remnant in the abdomen using a circular transanal stapling device or in some cases a handsewn anastomosis will be performed. The reconstruction is rarely protected by a temporary diverting loop ileostomy.

#### IPAA

The IPAA can be performed both as an open or, preferably as a laparoscopic or robotic procedure. A trans-anal minimal invasive method (TaTME) may be used to facilitate proctectomy [16]. A pouch is constructed with the last part of the distal ileum and then anastomosed to the anal canal, or to a small rectal remnant, usually 1–2 cm in length, often referred to as the rectal cuff. The anastomosis is often created with a transanal circular stapling device but can also be hand sutured if needed. In most, but not all cases, the reconstruction is protected by a temporary loop ileostomy. In those instances, the loop closure, when bowel continuity is restored, will be considered the index operation in the study. Technical aspects of the creation of the pouch was at the discretion of the individual surgeon, however recorded in the clinical report form.

#### Controls

In order to obtain a comprehensive overview of all UC patients that undergo colectomy, patients that decline reconstruction or those eligible only for one method of reconstruction (e.g. only IPAA due to refractory proctitis) will be asked to participate as controls.

#### Failure

Patients converted from IRA to IPAA or from either reconstruction to a permanent ileostomy will be analyzed in an “intention-to-treat” manner.

### Data collection

#### Instruments

General QoL will be assessed with the SF-36 form [17], IBD specific QoL with the SHS [18], bowel function with the Öresland score [19], female sexual function with the FSFI-6 [20], male sexual function with IIEF-5 [21]. In addition to these validated forms questions on whether or not the patients are satisfied with their choice of operation, would choose it again and recommend it to others and questions on fertility and reproduction will be asked. Early complications will be measured according to the Clavien-Dindo scale [22].

Data will also be obtained on smoking status, UC medication, age, indication for colectomy (chronic active disease/acute flare/dysplasia), BMI, endoscopic status in pouch/rectum, reoperations and for each operation: operation technique, operative time, bleeding, perioperative complications and hospital stay.

The data is collected and managed using REDCap (Research Electronic Data Capture) [23, 24] hosted at the Karolinska Institute, Stockholm, Sweden.

#### Time frame

**Fig. 3:**
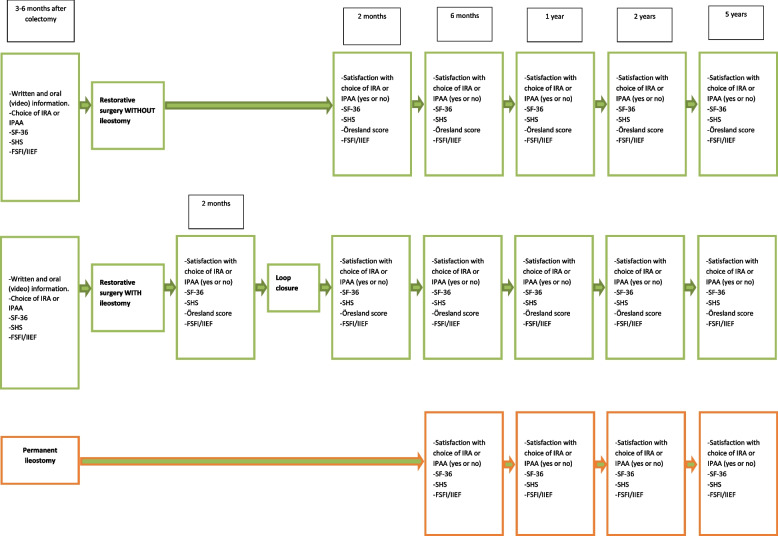
Timeline describing the collection of QoL and functional score questionnaires in the study population

Baseline data will be collected after colectomy for all patients. The reconstructed patients will then be followed at 2 months, 6 months, 1 year, 2 years and 5 years. Those patients that receive a loop ileostomy at reconstruction will also be followed 2 months after reconstruction before closure of the loop. For those patients the loop closure is considered the index operation. The patients that choose to (or are deemed to) keep their end ileostomies will be followed at 6 months, 1 year, 2 years and 5 years after colectomy (Fig. 3). The recruiting of patients started in March 2017.

### Statistical analysis

Analysis will be conducted according to the intention-to-treat principal. The primary outcome satisfaction, a proportion, will be compared with chi-square test. For the primary outcome two-tailed tests will be applied since the null hypothesis is that there is no difference between the two reconstructive methods. Secondary outcomes assumed to be normally distributed will be compared between the two study arms using t-tests. Functional and QoL variables will be analyzed with mixed model ANOVA analysis. Time to failure will be analyzed with multivariate cox-regression analysis. Kaplan–Meier curves will be constructed and compared with log-rank tests. Repeating events, such as reoperations, will be analyzed with multivariate Poisson-regression.

There is no data and safety monitoring committee since all treatment is according to established practice. We do not plan to perform any interim analysis.

From a statistical point of view our choice of primary endpoint is not optimal because we have no reason to expect any difference between the two study arms. Still, to our opinion, this is the most important outcome measure.

## Discussion

Reconstruction after colectomy is a morbidity-associated as well as a resource-intensive activity with the sole purpose of enhancing function, QoL and patient satisfaction. The actual disease has already been treated with the colectomy. Hence it is crucial to provide patients and care givers with the best possible information on the risks and benefits of each reconstructive treatment. They should be informed of what to expect if they choose to keep their end ileostomy permanently. There are no prospective head-to-head comparisons between IRA and IPAA in UC patients published.

The aspects to consider when evaluating a reconstruction after colectomy are early as well as late complications of the reconstruction, bowel function as well as urogenital and sexual function, quality of life (QoL), patient satisfaction as well as risk of failure and/or rectal cancer. There are also patient preferences which must be considered.

The IRA is easier to perform and does not require pelvic dissection, but instead the preserved rectum demands continuous topical medication and the risk for rectal cancer remains [25-27]. Hence, patients with an IRA needs to be surveilled for cancer and patients with increased colorectal cancer risk (i.e. patients with previous colorectal cancer or primary sclerosing cholangitis (PSC) are not considered suitable for IRA [25].

Early complications generally refers to complications within 30 days of surgery and they are most often graded according to the Clavien-Dindo scale [22]. In most studies a Clavien-Dindo score of 3b (a complication requiring intervention under general anesthesia) is considered to be of clinical significance. For IPAA, Clavien-Dindo scores of 3b or worse are reported in between 10–18% of patients [28, 29]. The only available report on early complications after IRA reports a 12.4% incidence of Clavien-Dindo 3a (a complication requiring intervention under local/regional anesthesia) or worse [30].

Considering late complications, pouchitis for IPAA is reported in between 36–48% of cases [31-33] with increasing cumulative numbers over time and up to 70% at 20 years follow up [33]. Some degree of proctitis at some point is reported in between 59–76% of IRA cases [34, 30]. There is to our knowledge only one report that specifies reoperation rates after IPAA and none on IRA. In the only available publication Wasmuth et al*.,* reports a reoperation rate of 33% after a mean follow-up of 10 years and estimates that 52% of IPAA patients will have had at least one reoperation after 20 years [35].

Regarding bowel function, the main issues are continence, number of bowel movements, need to evacuate the bowel during nighttime and urgency to evacuate the bowel. For IRA, between three and six bowel movements per 24 h have been reported [30, 36-39] while for IPAA between five and seven bowel movements are reported [30, 40]. Need for night-time evacuation have been reported in 13–41% of IRA patients and 53% of IPAA patients. Among IRA patients, faecal incontinence is reported in 5% of patients and seepage or need for protective pads for 11–19% of patients [36, 39]. For the IPAA patient these figures are 14% and 32–39% [39, 41] Urgency is reported in 33–68% of IRA patients compared with 16–23% of IPAA patients [36, 39, 42, 43].

The pelvic dissection associated with IPAA constitutes a risk for impaired sexual function and impaired fecundity. Sexual function is described and compared in different ways in IPAA studies and the results are difficult to compare [44-49]. In female patients with an IPAA, sexual dysfunctions is reported in up to 50% of cases [44, 45] using validated sexual function forms, e.g. the FSFI [20, 21, 50, 51]. Fewer problems are reported among male IPAA patients and there are even reports of improved sexual function after surgery [52, 53]. There is, to our knowledge, very little published on the impact of IRA on sexual function [25]. However, Moreira et al*.,* reported a tendency towards better sexual function among IRA patients compared to IPAA patients [39]. The actual reproductive rate is referred to as fecundity. Female UC patients are reported not to have impaired fertility compared to the general population [54]. Fecundity in female UC patients dropped from normal levels to 0.2 after IPAA [55] and infertility rates have been reported to increase from 20 to 63% after IPAA in a meta-analysis consisting of studies with both UC and FAP patients [56]. There is a reasonable hope that laparoscopic surgery may reduce the functional problems after IPAA but that remains to be further investigated [57, 58]. It was previously suggested that IRA does not reduce fecundity/fertility [59, 60] In contrast, Challine and colleagues recently compared fecundity between females subjected to IRA and IPAA and found no significant difference between the two [61]. This was in turn contradicted by the findings from the Swedish national cohort published by Druvefors [62]. In the French cohort they found reduced fecundity among females subjected to open surgery compared to laparoscopic regardless of IPAA or IRA [61] while the number of laparoscopic procedures was to few to render further analysis in the Swedish material [62].

In reconstructive surgery, the goal is to improve quality of life (QoL). Still, it is not obvious how to assess and compare QoL in surgery for UC [63]. In studies comparing IPAA to end ileostomy, an improved body image was reported among the IPAA patients but otherwise similar satisfaction and QoL [64-67]. There are several studies investigating how QoL develops over time in IPAA patients. It appears that the QoL of the IPAA patients is somewhat impaired in the first months after surgery but then improves [68, 69] and the QoL of the IPAA patients appears to be good in the long-term [42, 70]. Available studies comparing QoL in IRA to IPAA reports similar [15, 71] QoL between the two procedures but one study reported more urgency affecting work and dietary restrictions among the IRA patients [72].

Failure, defined as excisions or permanent deviation, is reported in between 4–9% of IPAA cases at 5 years and 7–19% at 10 years [30, 73-78] and reports with longer follow up indicates a continuing annual failure rate of around 2% even after 15 years [79]. The reported failure rates for IRA are 10–16% at 5 years and 24–31% at 10 years [30, 34, 39, 80, 81]. One should remember that after excising a poorly functioning IRA it is possible to construct an IPAA. However, the options after excising an IPAA are either a redo IPAA or an end ileostomy [82].

The obvious limitation with our study design is the lack of randomization. As mentioned in the introduction we have made an attempt to randomize between IRA and IPAA but after receiving detailed information, patients decline randomization and insist on choosing a reconstructive method themselves, with similar numbers opting for IRA and IPAA. We do not consider it a risk, but a fact, that some degree of selection bias will occur as some patients are more concerned regarding pelvic surgery, and its possible consequences, while others are more concerned by the need of anti-inflammatory medication, endoscopic surveillance and the risk of rectal cancer. We aim to minimize such effects with the provision of standardized information whenever possible. Because this is a multicenter study the same consultant cannot present the oral information to every patient. Instead, all patients will be shown the same information video by the consultant IBD Surgeon.

We do, however, see an upside to the lack of randomization. It is of interest to see what patients choose when presented with standardized information and if there are any demographic patterns in the choice of reconstructive method. Furthermore, we do not think that IRA will necessarily be a better choice than IPAA but rather it will be equally good in the selected cohorts eligible for either restorative procedure. Patient involvement may also increase the chance of a favorable outcome.

Another question that we hope to address is if there are any differences in the outcome of IPAA between the patients that were eligible for both IRA and IPAA and the patients that had IPAA as their only restorative option, i.e. will the level of proctitis affect the outcome of IPAA.

The multinational setting of the study will also allow for detection of possible differences in the attitude towards functional outcomes and complication patterns between the Swedish and English UC population. It will also improve the external validity of our study.

## Conclusion

Because we have failed to enroll patients in an RCT we believe this is the best available way to compare the outcomes between IRA and IPAA. This design will also provide a good overview of the entire UC population that required colectomy.

## Trial status

As of September 28, 2022, we have enrolled 37 IRA patients and 11 IPAA patients in the study arms and 23 IPAA and 18 ileostomy patients in the control arms.

## Data Availability

Not applicable, no data, only protocol.

## Abbreviations

IBD: Inflammatory bowel disease
UC: Ulcerative Colitis
CD: Crohn’s disease
IBD-U: Inflammatory Bowel Disease-Unclassified
QoL: Quality of Life
CI: Continent ileostomy
IPAA: Ileal pouch anal anastomosis
IRA: Ileorectal anastomosis
PSC: Primary Sclerosing Cholangitis
RCT: Randomized controlled trial
SF-36: Short form 36 item
SHS: Short health scale
FSFI: Female Sexual Function Index
IIEF: International Index of Erectile Dysfunction
TaTME: Trans anal total mesorectal excision
